# Analysis of colony phase variation switch in *Acinetobacter baumannii* clinical isolates

**DOI:** 10.1371/journal.pone.0210082

**Published:** 2019-01-04

**Authors:** Irfan Ahmad, Nabil Karah, Aftab Nadeem, Sun Nyunt Wai, Bernt Eric Uhlin

**Affiliations:** 1 The Laboratory for Molecular Infection Medicine Sweden (MIMS) and The Department of Molecular Biology, Umeå University, Umeå, Sweden; 2 Institute of Biomedical and Allied Health Sciences, University of Health Sciences, Lahore, Pakistan; Universidad Nacional de la Plata, ARGENTINA

## Abstract

Reversible switching between opaque and translucent colony formation is a novel feature of *Acinetobacter baumannii* that has been associated with variations in the cell morphology, surface motility, biofilm formation, antibiotic resistance and virulence. Here, we assessed a number of phenotypic alterations related to colony switching in *A*. *baumannii* clinical isolates belonging to different multi-locus sequence types. Our findings demonstrated that these phenotypic alterations were mostly strain-specific. In general, the translucent subpopulations of *A*. *baumannii* produced more dense biofilms, were more piliated, and released larger amounts of outer membrane vesicles (OMVs). In addition, the translucent subpopulations caused reduced fertility of *Caenorhabditis elegans*. When assessed for effects on the immune response in RAW 264.7 macrophages, the OMVs isolated from opaque subpopulations of *A*. *baumannii* appeared to be more immunogenic than the OMVs from the translucent form. However, also the OMVs from the translucent subpopulations had the potential to evoke an immune response. Therefore, we suggest that OMVs may be considered for development of new immunotherapeutic treatments against *A*. *baumannii* infections.

## Introduction

*Acinetobacter baumannii* has emerged as one of the most common causative agents of a wide range of nosocomial infections, such as ventilator-associated pneumonia, septicaemia, skin and soft tissue infections, and catheter-associated urinary tract infections [1–3]. Community-acquired *A*. *baumannii* infections have also been reported, mainly in patients with underlying comorbidities, including pulmonary disorders, diabetes mellitus, alcohol use disorder and cancer [4]. *A*. *baumannii* acquired the status of “key human pathogen” in the late 20^th^ century because of the frequent isolation from patients all over the world. However, the capacity of producing discernible toxins with virulence potential, as found in many other bacterial pathogens, has not been reported in *A*. *baumannii*. Our current understanding of *A*. *baumannii* virulence and pathogenesis suggests that a ‘persist and resist’ strategy is a major virulence feature [5]. Like many other Gram negative bacteria, *A*. *baumannii* secretes outer membrane vesicles (OMVs). OMVs from *A*. *baumannii* are equipped with phospholipases and exhibit haemolytic and leucocytic potential [6]. Surface proteins of *A*. *baumannii* OMVs were shown to elicit pro-inflammatory immune response in epithelial cells and neutrophil infiltration in a lung infection mice model [7].

*A*. *baumannii* has a remarkable capability to survive in unfavourable conditions, e.g. during exposure to disinfectants, antibiotics or immune cells [8,9]. The ability to resist antibiotics in *A*. *baumannii* is typically associated with a frequent horizontal uptake of antibiotic resistance genes. However, the actual mechanisms of persistence and resistance to disinfectants remain less known. Getting insights into the morphological and physiological features, and exploring the genetic contents, of *A*. *baumannii* clinical isolates would be a relevant starting point towards elucidating the persistence and resistance strategies of this opportunistic pathogen [10,11]. Colony switch between opaque and translucent forms is an interesting morphological feature that has been recognised recently in *A*. *baumannii*. One of the *A*. *baumannii* clinical strains, AB5075, produced either translucent or opaque colony morphotypes on agar plates [12]. The two phase variants could reversibly switch their phenotypes, from opaque to translucent or translucent to opaque. The opaque and translucent phase variants of AB5075 were shown to exhibit significant phenotypic differences in cell morphology, surface motility, biofilm formation, antibiotic resistance and in the virulence to the *Galleria mellonella* waxworm infection model and in a lung infection mice model [12]. The opaque variants were reported to exhibit better resistance to antibiotics, disinfectants, reactive oxygen species, lysozymes, the cathelin-related antimicrobial peptide CRAMP and to be covered with thicker capsule coating [13]. The precise molecular mechanism of colony switching remains to be elucidated. However, there is some evidence that yet unidentified diffusible extra-cellular signal(s) may induce phase switch in a density dependent manner [12].

The attempt to identify underlying mechanisms of phenotype switching unravelled the role of aTetR-type transcriptional regulator, ABUW_1645 in colony switching. The over expression of ABUW_1645 locked the cells into a translucent subpopulation. However, deletion of ABUW_1645 did not abolish the switching phenotype [13]. Also, deletion of the genes encoding resistance-nodulation-cell division (RND)-type efflux system *arpAB* significantly decreased phase variation in the opaque to translucent direction, but had little to no effect on phase variation in the translucent to opaque direction. Moreover, mutations inactivating this RND system were pleiotropic and resulted in altered surface motility, aminoglycoside resistance, and virulence in a *G*. *mellonella* waxworm model [14]. In contrast, the deletion of OmpR orthologue ABUW_0257 activated phase switching in the opaque variant to inter convert at an extremely high frequency. Similarly, mutations in the sensor kinase *envZ* gene also activated phase variation from the opaque to translucent variant. In addition, OmpR and EnvZ in *A*. *baumannii* strain AB5075 were required for virulence of the opaque variant in the *G*. *mellonella* infection model [15]. Based on efficiency to colonize to lungs of infected mice, opaque variants were proposed to be “virulent” and translucent variants were proposed to be “a virulent subpopulation of AB5075 [13].

Here, we investigated clinical isolates of *A*. *baumannii* for switching from and to opaque and translucent subpopulations, and compared these subpopulations with regard to their surface morphology at single cell level, biofilm formation, virulence to *Caenorhabditis elegans*, and production of OMVs. Furthermore, the ability of *A*. *baumannii* and secreted OMVs to induce an immune response in human macrophages is presented.

## Materials and methods

### Bacterial strains used

Fifty-four clinical isolates of *A*. *baumannii* collected in Pakistan between 2013 and 2015 were tested for their opaque/translucent colony morphology. Based on their multilocus sequence types, six isolates were selected for further assays (Table 1), including three strains from the Pakistanis collection, Ab-Pak-Pesh-22 belonging to sequence type 23 (ST23), Ab-Pak-Pesh-37 (ST1106), and Ab-Pak-Lah-14 (ST1), two strains from our Swedish collection of *A*. *baumannii*, A095 (ST2) and A100 (ST1) [19], and the hyper virulent *A*. *baumannii* reference strain AB5075 (ST1) [12]. All the strains were routinely grown on LB agar plates at 37°C.

**Table 1:** *Acinetobacter baumannii* strains used in the study.

Strain	Source, place and date of isolation	MLST typing	Reference
Ab-Pak-Pesh-22	Tracheal Wash, CMH Peshawar, Pakistan, 2014	ST23	a) This work
Ab-Pak-Pesh-37	Tracheal Wash, CMH Peshawar, Pakistan, 2014	ST1106	a) This work
Ab-Pak-Lah-14	Tracheal wash, CMH Lahore, Pakistan, 2013	ST1	a) This work
A095	Feces, Stockholm, Sweden, 2013	ST2	[19]
A100	Not known, Skåne, Sweden, 2013	ST1	[19]
AB5075	Tibia/osteomyelitis, USA, 2008	ST1	[27]

a) Whole genome sequence data deposited at DDBJ/ENA/GenBank under the BioProject accession number: PRJNA482499.

### Colony phase variation analysis

The colony phase variation assay was performed as previously described with slight modifications [12]. Briefly, single colonies from the overnight cultures were grown in LB broth to an OD of 1.8. Serial dilutions of the LB broths were made and 100 μl allocations were re-plated on LB agar plates. After overnight growth, the total number of viable cells per ml was determined. The morphology of opaque or translucent colonies was reviewed by stereo light microscopy. The number of these two phase variants of colonies was counted only on plates with ≥200 colonies/plate.

### Biofilm formation analysis

The capacity of *A*. *baumannii* isolates to produce biofilms was assessed in 96-well microtiter plates. Bacterial suspensions, with an OD_600_ of 0.1, were prepared using the LB broth. Then, 200 μl of the suspensions were inoculated in triplicates into a 96-well flat bottomed polystyrene microplate and incubated at 37° C or 30° C for 24 hour under static conditions in a moist chamber. Following incubation, the wells were washed gently three times with sterile water, and then fixed by warming on hot plate for 30 sec. To stain biofilms, 200 μl of 1% crystal violet solution in water was added into each well, incubated at room temperature for 30 min. The dye was discarded and the wells were washed twice with distilled water, dried, and the stain was solubilized by 150 μl of 5% acetic acid. The optical absorbance was read at 570 nm using a microtiter-plate reader. Each experiment was repeated three times.

### *C*. *elegans* fertility analysis

Virulence of the *A*. *baumannii* clinical isolates was assessed using the *C*. *elegans* fertility assay as a model system [16]. The *C*. *elegans* eggs were hatched in M9 medium, grown to stage L1 and arrested overnight at 16°C to physiologically synchronise the nematodes. The L1 worms were cultivated in *Escherichia coli* OP50 lawns to L4 stage. The fertility assay was performed by inoculating a single L4 nematode per each Nematode Growth Media (NGM) plate containing a lawn of the bacterial strain under investigation. The plates were incubated at 30° C. Over a period of 5 days, adult nematodes were transferred daily to a fresh plate seeded with the same strain of bacteria. To determine fertility, nematode progeny were counted daily, 48 hour after parent removal. Six independent replicates were performed with each bacterial strain, and fertility assays were performed in triplicate. Mean values of the differences between strains are reported. *Vibrio cholerae* strain C6706*luxO*^*C*^ was used as positive control for virulence whereas *E*. *coli* OP50 was used as negative control for virulence in *C*. *elegans*.

### OMV preparation

OMVs were isolated from bacterial culture supernatants as described previously [17]. Briefly, 1 liter of each bacterial culture, grown in LB broth at 37°C for 16 h, was centrifuged at 5000 x *g* for 30 min at 4° C. The supernatant was filtered through a 0.2-μm pore size sterile Minisart High Flow syringe filter (Sartorius Stedim) and ultracentrifuged at 100,000 x *g* for 2 h at 4°C in a 45 Ti rotor (Beckman). The vesicle pellet was resuspended in 20 mM Tris-HCl pH 8.0 buffer and the suspension was used as the crude OMV preparation. The OMV preps were analysed by SDS-PAGE and by atomic force microscopy. The Bicinchoninic Acid (BCA) Assay kit (Thermo Scientific Pierce, Rockford, IL) was used to measure the samples’ total protein content.

### Atomic Force Microscopy

The OMVs were imaged by Atomic Force Microscopy (AFM) as described previously [18]. Briefly, 10 μl of the OMV samples were placed onto freshly cleaved mica (Goodfellow Cambridge Ltd., Cambridge, United Kingdom). The samples were blot dried and desiccated prior to imaging. Imaging was done on a Multimode 8 Nanoscope AFM equipment (Digital Instruments, Santa Barbara) using Tapping ModeTM. A silicon probe was oscillated at its resonant frequency of approximately 300 kHz, selected by the Nanoscope software. Images were collected in air at a scan rate of 0.8–1.5 Hz, depending on the size of the scan and the number of samples (256 or 512 samples/image). The final images were plane fitted in both axes and presented in a surface plot of the height mode.

### Scanning electron microscopy

For scanning electron microscopy, 5 μl of the bacterial suspensions in PBS (OD_600_ of 1), from an overnight plate culture, were spotted onto LB agar plates. The plates were incubated at 37°C for 24 hours. Pieces of agar containing bacterial colonies were removed, fixed overnight at 4° C with 2.5% glutaraldehyde in 0.1 M sodium cacodylate, dehydrated in graded series of ethanol, and coated with 5 nm gold/palladium. The bacterial cells morphology was analyzed by field-emission scanning electron microscope (Carl Zeiss Merlin FESEM) using secondary electron detectors at accelerating voltage of 4 kV and probe current of 50–100 *p*A.

### Co-culture of murine macrophages with *A*. *baumannii*

Murine macrophage cell line RAW 264.7 (ATCC® TIB-71 ™) were grown on coverslip (5x10^4^ cells) overnight in DMEM media supplemented with 10% FBS and 1% penicillin-streptomycin. Bacteria from overnight cultures of *A*. *baumannii* were washed twice and re-suspended in phosphate-buffered saline (PBS). Re-suspended *A*. *baumannii* was added to the RAW 264.7 cells at a multiplicity of infection (MOI) of 100:1. Co-cultures were incubated for 4 h and for 4 h at 37°C with 5% CO_2_. Cell viability was determined by trypan blue exclusion.

### Live cell confocal microscopy

RAW 264.7 cells were seeded on a coverslip (5x10^5^ cells) and incubated overnight at 37°C in 5% CO_2_ to allow adherence. The following day, cells were treated with PKH2 labelled OMVs (100μg) for 4 h. For staining the cell nuclei, Hoechst 33342 (1 μM) was added 5 min before the end of the 4-h incubation time. Subsequently, cells were washed with DMEM. Finally, DMEM medium containing 10% FBS was added to the cells and visualized live under Nikon EZ-C1 confocal microscope (Nikon, Japan), using 40 X /1.4 plan Apo λs lens. The λ_ex_ = 405nm was selected for Hoechst 33342 and λ_ex_ = 561 nm for OMVs. Images were captured and processed using the NIS-Elements (https://www.nikoninstruments.com/Products/Software) and ImageJ (https://imagej.net/) software.

### Immunohistochemistry analysis

After 4 h infection, cells were fixed (4% PFA, 30 min) and permeabilized (0.25% Triton X in PBS, 20 min). Cells were incubated with anti-NFKB primary antibodies (Santa Cruz, sc-81932, 1:100 dilution) in 10% FBS/PBS overnight at 4° C, and then with secondary Alexa-488 or Alexa-568 conjugated antibodies (1:200 in 10% FBS/PBS, Molecular Probes, Eugene, OR, USA, 1 h), counterstained with DAPI (Sigma) and examined by NikonEZ-C1 confocal microscope (Nikon, Japan), using 60 X /1.4 plan Apo λs lens. The λ_ex_ = 405nm was selected for DAPI and λ_ex_ = 488 or λ_ex_ = 561 nm for NFKB. Images were captured and processed using the NIS-Elements (https://www.nikoninstruments.com/Products/Software) and ImageJ (https://imagej.net/) software.

### Statistical analysis

Graph Pad Prism version 7.09 was used to construct graphs and analyse data regarding phase switching frequencies, biofilm formation and *C*. *elegans* progeny counts.

Quantification of cells was presented as a Mean±SEM. Groups were compared using paired t-test (Microsoft Excel). *p* values of <0.01 ** and <0.05 * were considered significant.

## Results

### Dynamics of opaque translucent switch in *A*. *baumannii* clinical isolates

A collection consisting of fifty-four clinical isolates of *A*. *baumannii* from Pakistan was tested for opaque/translucent colony morphology. All the 54 *A*. *baumannii* isolates produced a predominant opaque colony morphotype on the LB agar plates. However, a small fraction of the colonies, from each strain, appeared to be translucent. The fraction of translucent colonies was < 0.01% in all isolates. Six clinical isolates were further investigated for details in phenotypic alterations upon colony switching. The panel of isolates subjected to more detailed analysis of phase switching consists of *A*. *baumannii* Ab-Pak-Pesh-22 (ST23), Ab-Pak-Pesh-37(ST1106), Ab-Pak-Lah-141(ST1), two isolates from our Swedish collection of *A*. *baumannii*, A095(ST2) and A100 (ST1) [19] and the hyper virulent *A*. *baumannii* reference strain AB5075 (ST1).

**Fig 1:**
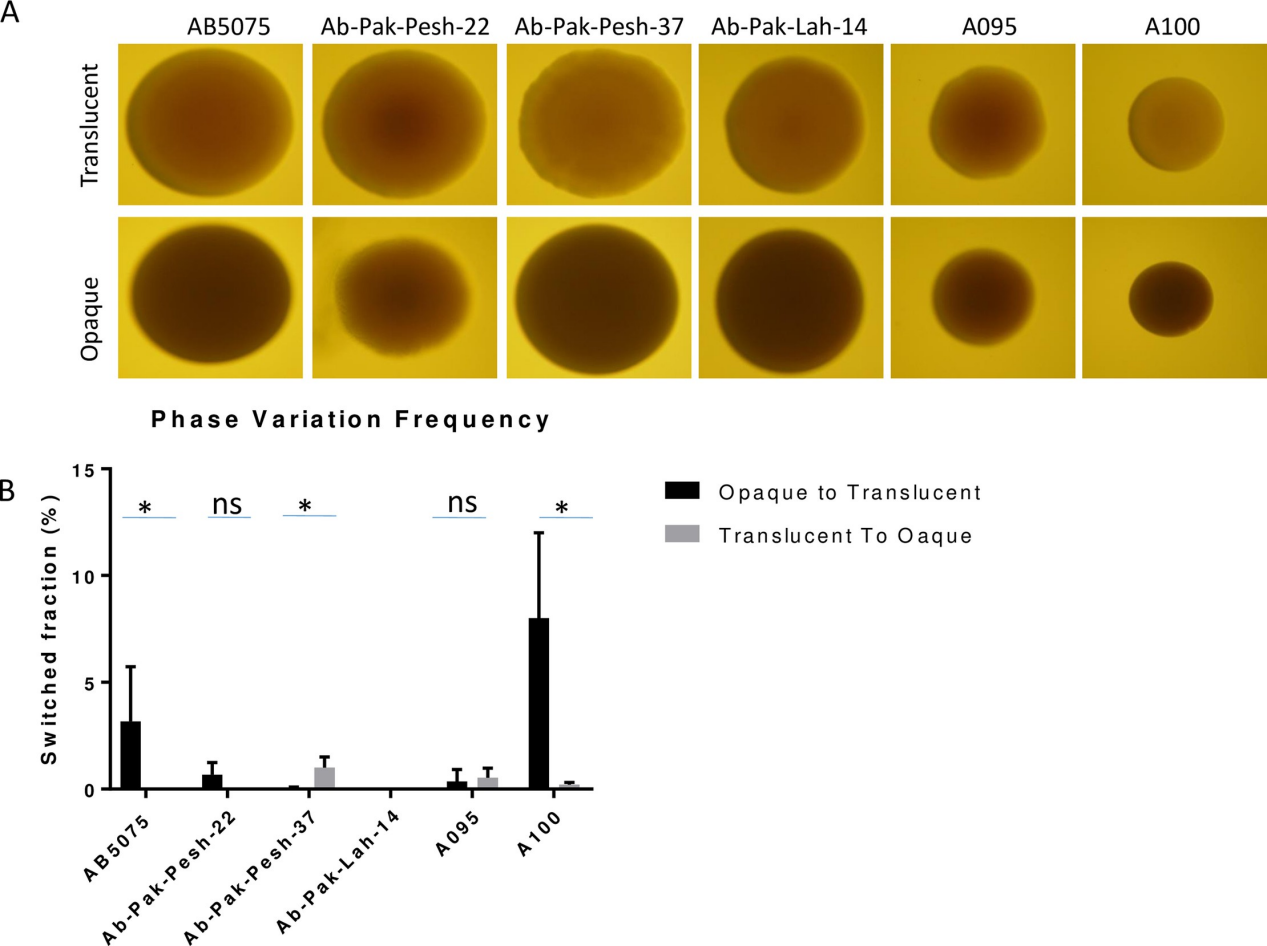
Opaque and translucent variants of *A*. *baumannii* clinical isolates. (A) Stereo microscopic images of opaque and translucent colonies, and (B) Phase variation switching frequency of *A*. *baumannii* clinical isolates. In order to compare switching frequencies the unpaired paired *t* test was performed. The difference is considered as statistically significant if *p* values is less than 0.05 and represented by “*”.

Stereo microscopic examination revealed a wide-range of strain-based differences in the intensity of the opaque and translucent colonies. Among the six isolates selected for further studies, A100 produced the most clearly distinguished translucent and opaque colonies (Fig 1A). In contrast, the difference between opaque and translucent colonies was less pronounced particularly in case of Ab-Pak-Pesh-22, but also Ab-Pak-Pesh-37 and A095 differed somewhat when compared to AB5075 and A100 (Fig 1A). Similarly, the switching frequency, from opaque to translucent or from translucent to opaque, was highly variable and entirely depended on the strain under investigation (Fig 1B). The Swedish isolate A100 switched from opaque to translucent at a significantly higher frequency (8.02± 1.90%) than the rest of the studied isolates. In general, the switching frequency from opaque to translucent was observed to be higher when compared to switching frequency from translucent to opaque (Fig 1B) [12]. Exceptionally, Ab-Pak-Pesh-37 switched from translucent to opaque at a significantly higher frequency (1.50±0.05%) than that of the opaque to translucent switch (0.05±0.02%).

### Visualization of opaque and translucent colonies at single cell level

**Fig 2:**
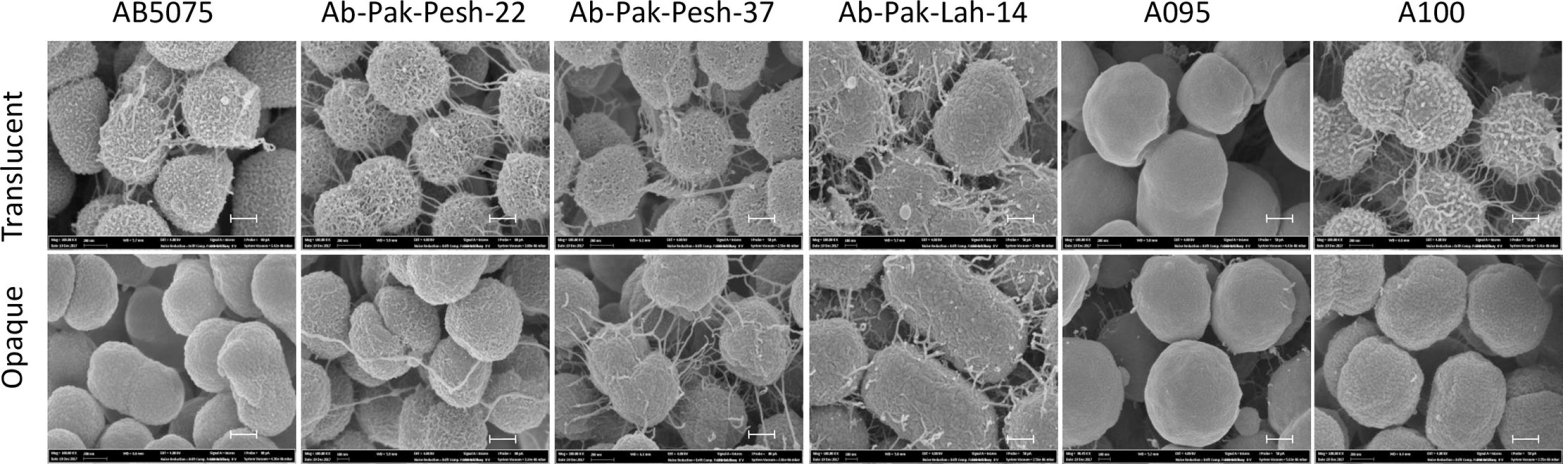
Scanning electron microscopic images of bacterial cells from opaque and translucent colonies of *A*. *baumannii* clinical isolates. Scale bar = 200 nm, Magnification 100,000x.

Scanning electron microscopy was performed to visualize the opaque and translucent variants at the single cell level. Overall, the cell surface of *A*. *baumannii* from opaque colonies appeared to be smooth whereas cells sampled from translucent colonies appeared to be rough and containing spinal shape appendages on the surface (Fig 2). However, the surface of *A*. *baumannii* A095 appeared smooth in case of both variants (Fig 1A). Also, the network of extracellular pili like structures tended to be denser among cells from translucent colonies except for isolate A095, which did not produce pili like appendages.

### Translucent colony subpopulations of *A*. *baumannii* form more pronounced biofilm in comparison with the opaque counter parts

**Fig 3:**
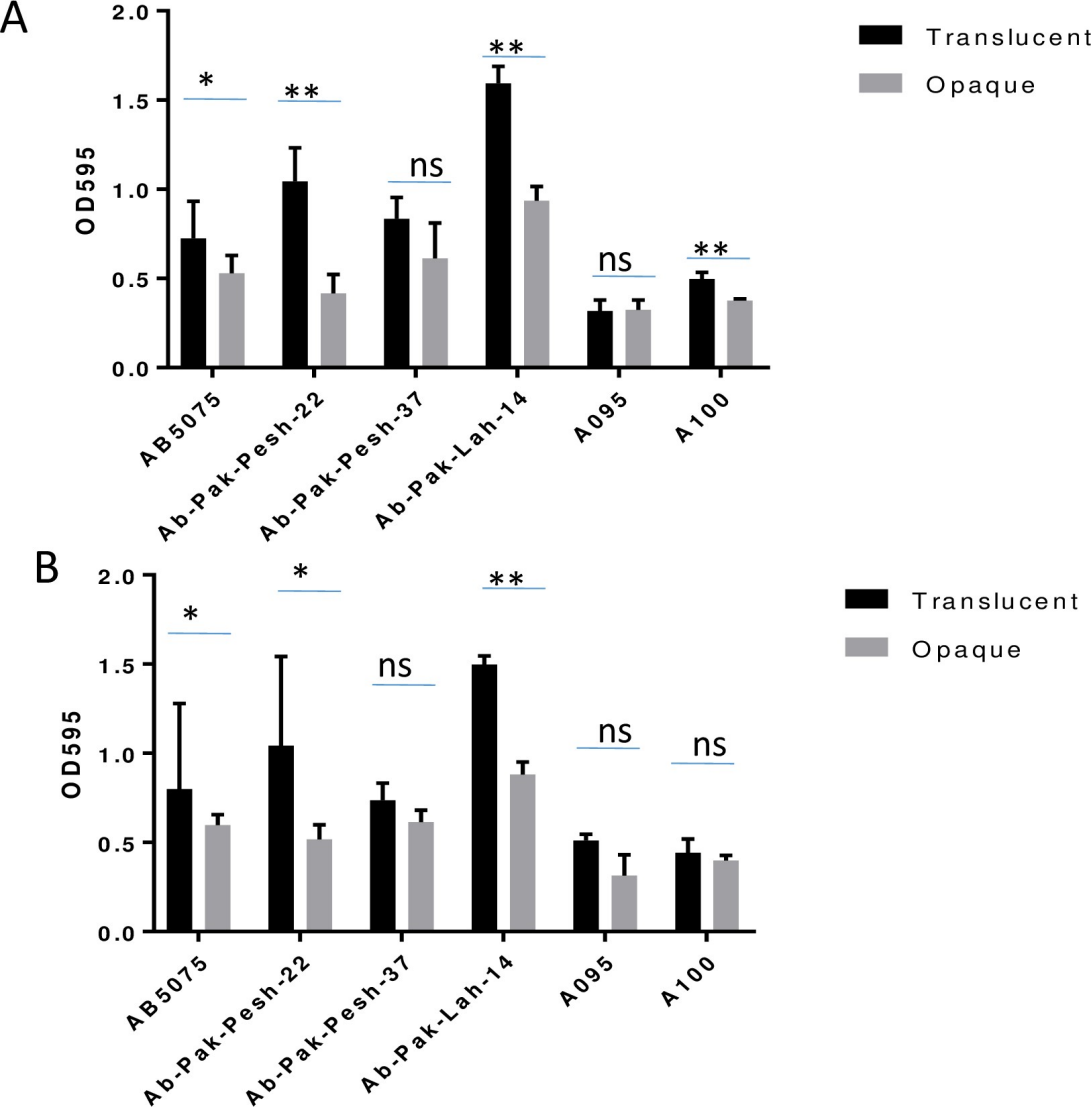
Biofilm formation capability of opaque and translucent colony variants. Biofilm formation after 24 hours at (A), 37° C, and (B) 30° C. Bars represent Mean± SD of OD_595_ values. Difference in biofilm forming capability of opaque and translucent strains was estimated by two tail paired *t* test. The difference is considered as statistically significant if *p* values is less than 0.05. *p* value less than 0.05 is represented by“*”, *p* value less than 0.01 is represented by “**”. *p* value higher than 0.05 is represented by ns (non significant).

The translucent colony variants showed significantly higher biofilm forming capability than the opaque colony variants (Fig 3). Consistent with the absence of extracellular pili-like structures (Fig 2), the two colony variants of isolate A095 appeared to form little or no biofilms.

### Virulence features of opaque versus translucent subpopulations

**Fig 4:**
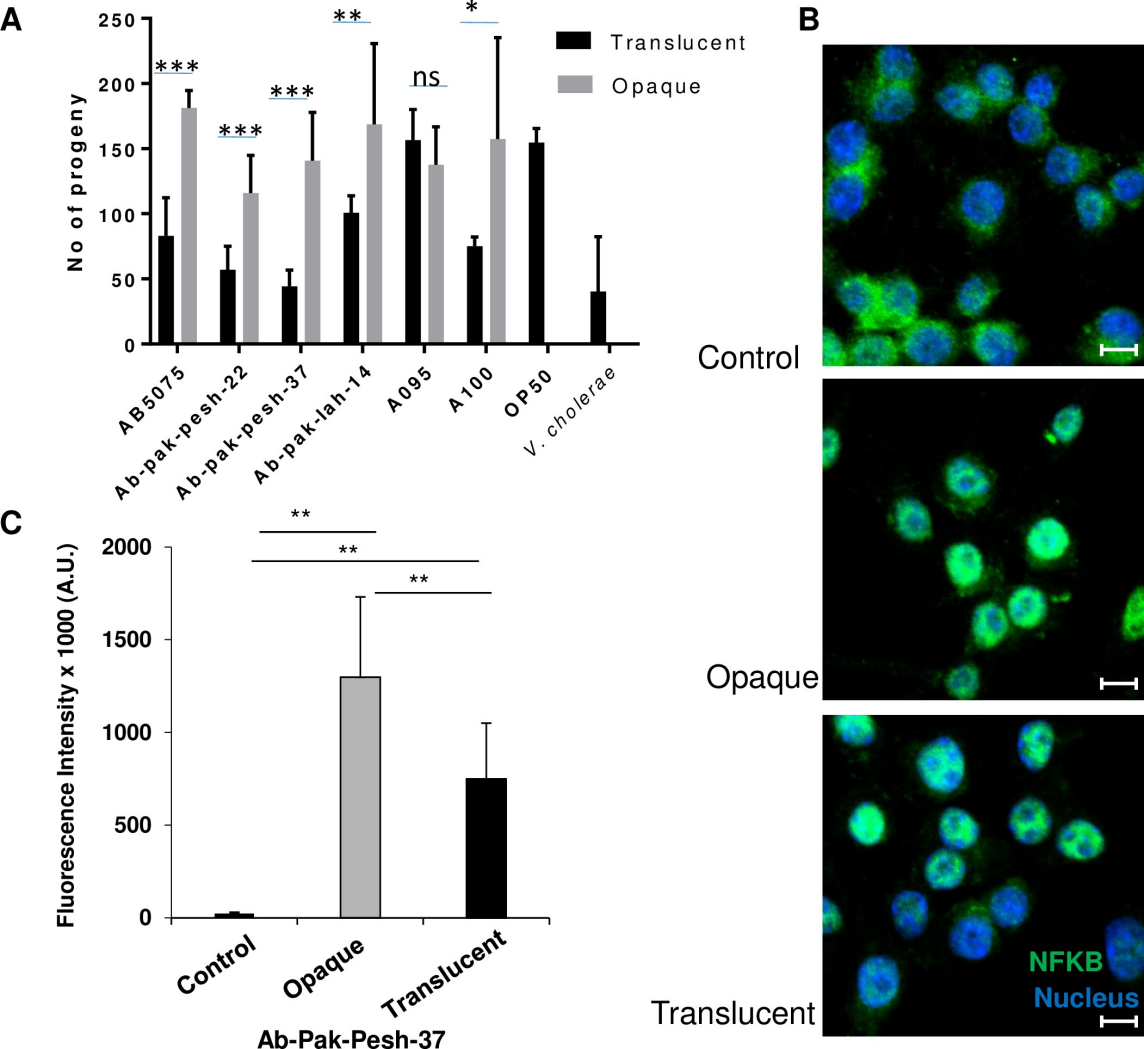
Virulence associated features of *A*. *baumannii* opaque and translucent variants. (A) Progeny count of *C*. *elegans* upon feeding with opaque and translucent variants of *A*. *baumannii*, *E*. *coli* OP50 and *V*. *cholerae* C6706*luxO*^*c*^, Bars represent Mean± SD of progeny counts. Difference in progeny count induced by opaque and translucent strains was estimated by two tail paired *t* test. The difference is considered as statistically significant if *p* values is less than 0.05. *p* value less than 0.05 is represented by“*”, *p* value less than 0.01 is represented by “**” and *p* value less than 0.001 is represented by “***”. *p* value higher than 0.05 is represented by ns (non significant). (B) NFKB nuclear translocation and activation in response to *A*. *baumannii* Ab-Pak-Pesh-37 opaque and translucent phase variants after 4 hours infection. Scale bar = 5μm, and (C) Quantification of NFKB staining intensity in cell nuclei (n = 20 cells). Groups were compared using paired t-test (Microsoft Excel). *p* values of <0.01 ** and <0.05 * were considered statisitically significant.

*C*. *elegans* and *G*. *mellonella* have proven to be efficient laboratory infection host models for monitoring putative virulence features bacterial pathogenesis. Using the *G*. *mellonella* model for *A*. *baumannii* infection, it has been shown that an opaque subpopulation of strain AB5075 exhibited higher virulence capability when compared to a translucent subpopulation [12]^,^. We were interested to evaluate *C*. *elegans* as an alternative model to monitor the pathogenesis potential of the opaque and translucent subpopulations of *A*. *baumannii*. In order to compare, the effect of opaque and translucent colony phase variants, a C. *elegans* fertility assay was performed as previously reported [16].The assay is quantitatively measuring the effect of the bacteria on the reproduction capability of an adult worm, monitored by a progeny count. Feeding of a translucent subpopulation of each isolate except *A*. *baumannii* A095 significantly reduced the progeny count of *C*. *elegans* in comparison with an opaque form of the same isolate (Fig 4A). This finding suggested that the translucent subpopulation exhibited a stronger effect on the fertility of *C*. *elegans*. In contrast, an opaque subpopulation was reported to be more virulent to *G*. *mellonella* and mice infection models [12]^,^. Therefore, our finding suggested that the effect of *A*. *baumannii* on *C*. *elegans* fertility was not due to the same virulence associated trait supposedly affecting the former infection models. Of note, *A*. *baumannii* Ab-Pak-Pesh-37 appeared to be the most efficient strain among the six isolates with respect to reduction in progeny of the worms.

As one test of how mammalian cells might be affected by the two phase variants, we further assessed the immunogenic potential of Ab-Pak-Pesh-37 in RAW 264.7 macrophages by quantifying nuclear accumulation of NFKB, 4 hours after infection. Interestingly, both opaque and translucent variants of Ab-Pak-Pesh-37 could elicit NFKB activation and nuclear accumulation (Fig 4B). However, NFKB activation by an opaque subpopulation was significantly higher as compared to the translucent counterpart (Fig 4B and 4C). This finding supported the notion that an opaque subpopulation may bes more virulent.

### OMV production by opaque and translucent colony variants

**Fig 5:**
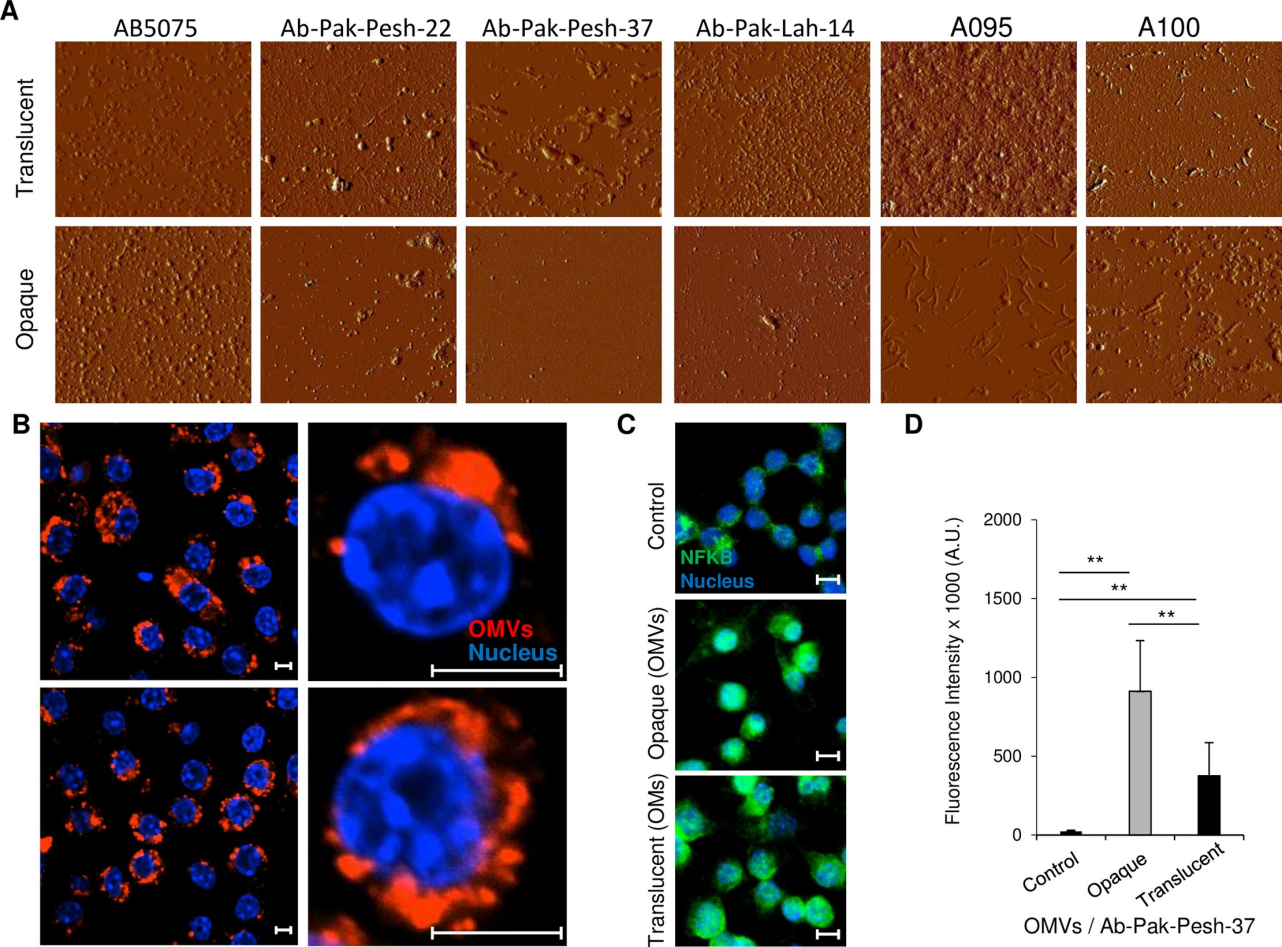
Outer membrane vesicles released by *A*. *baumannii* opaque and translucent variants. (A) Visualization of OMVs from opaque and translucent phase variants by atomic force microscopy, (B) Cellular uptake of micro vesicles (OMVs) isolated from *A*. *baumannii* Ab-Pak-Pesh-37 opaque (upper panel) and translucent (lower panel) phase variants. Scale bar = 5μm, (C) NFKB nuclear translocation and activation in response to OMVs isolated from *A*. *baumannii* Ab-Pak-Pesh-37. Scale bar = 5μm, and (D) Quantification of NFKB staining intensity in cell nuclei (n = 20 cells). Groups were compared using paired *t*-test (Microsoft Excel). Groups were compared using paired *t*-test (Microsoft Excel). *p* values of <0.01 ** or <0.05 * were considered statistically significant.

OMVs have recently emerged as a vehicle to transport virulence factors from bacterial pathogens to host cells. OMVs from opaque and translucent colony variants were isolated in order to compare the effect of colony phase switching on vesicles production. The number of OMVs per field imaged by atomic force microscopy was found to be higher when the OMVs were isolated from the translucent form of all *A*. *baumannii* isolates except for strain AB5075 (Fig 5A). Consistently, protein concentration of vesicle preparations from translucent colonies were significantly higher (1186±200 μg/ml) than protein concentrations of vesicles from opaque colonies (552±125 μg/ml) suggesting that translucent colonies released more vesicles than opaque colonies. In order to find out whether OMVs *per se* can be expected to play a role during infection process, the capability of OMVs from opaque and translucent forms of *A*. *baumannii* Ab-Pak-Pesh-37 to be internalized by macrophages was assessed. Interestingly, OMVs from both opaque and translucent bacteria were internalized at equal efficiency (Fig 5B). Also, both variants of OMVs possessed the capability to evoke NFKB activation after 4 hours of incubation (Fig 5C). However, the NFKB response generated by OMVs from opaque bacteria was significantly higher than that from the translucent form (Fig 5D). Importantly, the two variants of Ab-Pak-Pesh-37 or their OMVs did not elicit any detectable cytotoxicity to the cells during the 4 hours infection (S1 Fig).

## Discussion

Our assessment of colony phase variation switch in six clinical isolates of *A*. *baumannii* highlighted the occurrence of remarkable alterations in their phenotypic characters upon colony switching. These alterations are visible at the colony level, but can also be detected at the single cell level. The only exception was *A*. *baumannii* A100, where only minor alterations were observed upon its colony switching. *A*. *baumannii* Ab-Pak-Pesh-22 did not fully switch from opaque to translucent but other morphological characteristics were markedly altered upon partial switching. Similarly to previous studies, our findings suggested that switching from opaque to translucent was more efficient than the other way around [12]. However, *A*. *baumannii* Ab-Pak-Pesh-37, belonging to a rare strain type, behaved differently (Fig 1B). This strain showed a higher frequency of switching from translucent to opaque under the conditions used and it will be of interest to study if this behaviour is a common trait of the particular sequence type.

The results of co-culturing *A*. *baumannii* with murine macrophages (Figs 4B, 5C and 5D), were in line with earlier results of *A*. *baumannii* infection assays in mice or *G*. *mellonella* waxworm models [13, 15] and supported the proposal that opaque subpopulations of *A*. *baumannii* were more virulent than their translucent counterparts. Furthermore, screening a collection of 54 clinical isolates from patients in Pakistan revealed that the entire collection produced opaque colonies, with very small fractions of translucent subpopulations. These bacteria were isolated from a diverse range of specimens including sputum, bronchial wash and wounds. Similarly, opaque colonies were dominant in isolates collected from blood stream infections [13]. All these observations propose a dominant role of the opaque feature of *A*. *baumannii* in its human infection process.

*A*. *baumannii* clinical isolates did not kill the adult *C*. *elegans* during fertility assays. However, the translucent subpopulations efficiently reduced the fertility of *C*. *elegans* in contrast to the opaque subpopulations. This was an interesting observation in the context that the translucent phase was considered to be the avirulent form of *A*. *baumannii* clinical isolates [13]. It should also be noted that the translucent subpopulations appeared to forme more pronounced biofilms, as shown by our analysis (Fig 3) and in earlier studies [12]. Biofilm formation is an important fitness factor for survival in different environment settings [20, 21]. The *C*. *elegance* experiment was performed at a lower temperature (30°C) that occurs in environmental settings where many of the present *A*. *baumannii* isolates were obtained. Our results suggest that translucent subpopulations of *A*. *baumannii* might be more successful to persist under such environmental temperature conditions.

Like other Gram-negative rods, *A*. *baumannii* releases OMVs in the size range of 80 ± 20 nm in diameter with spherical shape [6]. OMVs have been shown to play a role as toxin carrying vehicles in many bacteria. *Acinetobacter* OMVs may transfer carbapenamase gene-containing plasmid DNA between *Acinetobacter* isolates and cause a decrease in extracellular β-lactam antibiotic concentrations via carabapenem-hydrolyzing class D β-lactamases (CHDL) and AmpC-like β-lactamases [22–24] metallo β-lactamase NDM-1[25]. Multi drug resistant strains of *A*. *baumannii* display higher outer membrane lipopolysaccharide expression, higher cytotoxicity, stronger innate immune responses and more virulence than the non-multi drug resistant strains [26]. We found that translucent phase variants produced a larger quantity of OMVs in comparison with opaque variants in case of most of our tested strains (Fig 5A). A thicker coating of extracellular capsule in opaque variants [13] might cause a reduced release of OMVs when compared to translucent cells where the outer membrane is relatively more exposed and open to release of membrane fragments and vesicles. Further characterisation of the actual components of the OMVs from different colony variants is needed. Being avirulent in the mice infection model, and based on our finding that their OMVs retain the potential to evoke an immune response, we suggest that translucent subpopulations of *A*. *baumannii* nevertheless could be good candidates for the development of an attenuated live vaccine and of new immunotherapeutic treatments against *A*. *baumannii* infections.

Analysis of the RNA transcriptome of *A*. *baumannii* opaque and translucent variants identified several candidate regulators of phase variation [13]. However, the questions “how and why colony switching occurs?” remain standing. Answering such questions should facilitate further understanding of the biology and pathogenicity of *A*. *baumannii* and might open the door towards novel approaches of management to improve prevention of *A*. *baumannii* infections.

## Supporting information

S1 Fig Phase switch variants of *A. baumannii* are not cytotoxic to cells. (PDF)
