# Supplementary material for: Analysis of colony phase variation switch in Acinetobacter baumannii clinical isolates
Source: PLoS One. 2019 Jan 4;14(1):e0210082. doi: 10.1371/journal.pone.0210082 (PMC6319719; doi:10.1371/journal.pone.0210082)
Supplement: S1 Fig — (PDF) [file pone.0210082.s001.pdf]

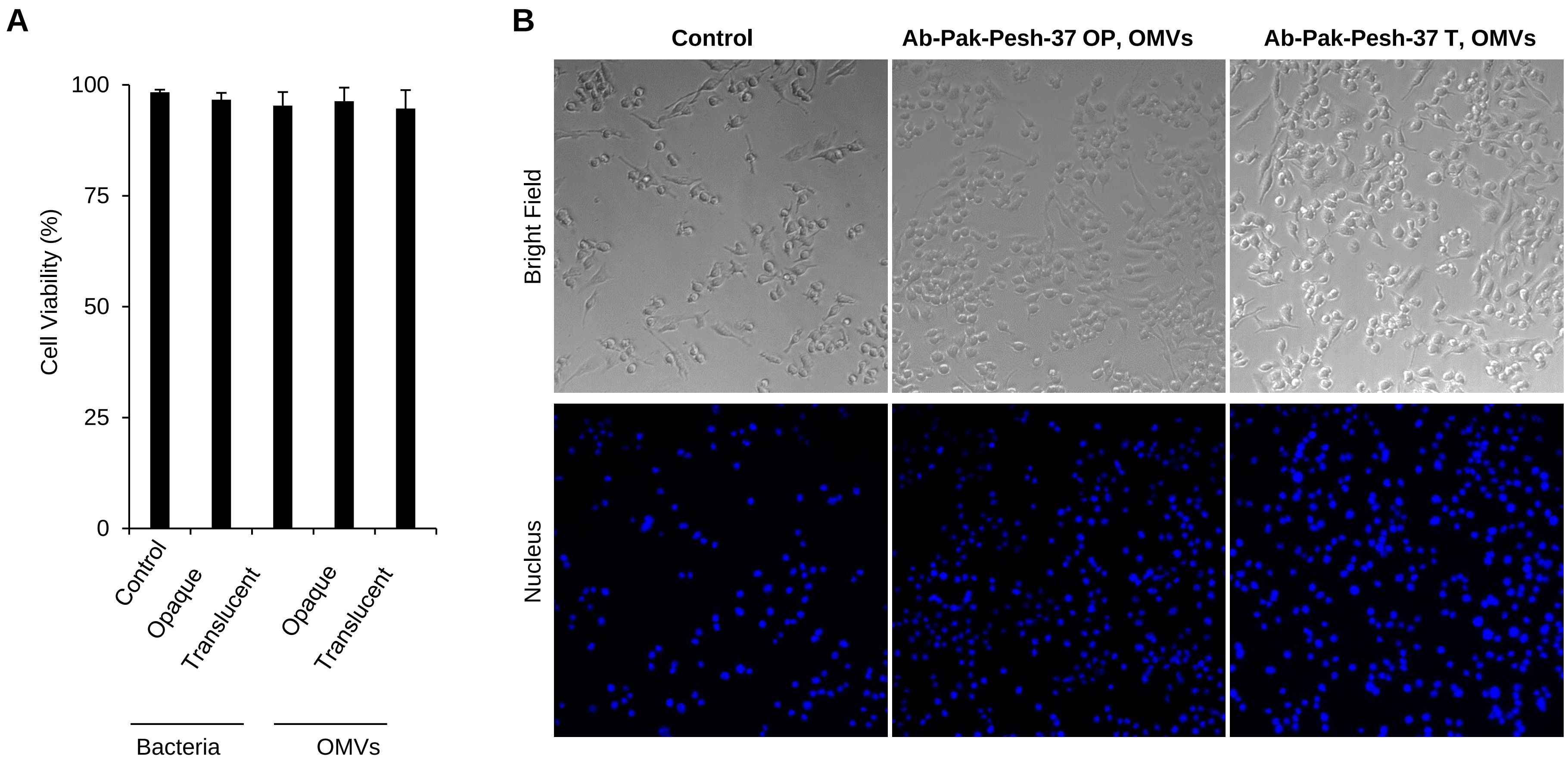

S1 Fig. Phase switch variants of *A. baumannii* are not cytotoxic to cells.

RAW 264.7 cells treated with *A. baumannii* opaque and translucent variants or outer membrane vesicles isolated from either of the variant had no effect on (A) cell viability as measured by number of Trypan Blue negative cells, or (B) cell morphology or nuclear condensation. OP = opaque, T = translucent.
